# Effect of Bile Acids Supplementation in Fatty Liver Hemorrhagic Syndrome, Production Performance, Physiological and Quality Characteristics of Laying Hen Eggs

**DOI:** 10.3390/ani14131910

**Published:** 2024-06-28

**Authors:** Wen Li, Yu Zhang, Jingyi Yang, Hao Xu, Ruiqi Ye, Jiale Wu, Mixia Cao, Chunfang Zhao, Bing Yang, Chang Liu, Lei Li

**Affiliations:** 1 College of Animal Science, Anhui Science and Technology University, Fengyang 233100, China; liwen5212022@163.com (W.L.); zhangyu20240610@163.com (Y.Z.); 13698864419@163.com (J.Y.); xuhaoank@163.com (H.X.); 15867745648@163.com (R.Y.); somewujialea@outlook.com (J.W.); cmx1578088@163.com (M.C.); zhaocf@ahstu.edu.cn (C.Z.); yangb@ahstu.edu.cn (B.Y.); 2 Anhui Province Key Laboratory of Animal Nutritional Regulation and Health, Fengyang 233100, China; 3 Key Laboratory of Quality & Safety Control for Pork, Ministry of Agriculture and Rural, Fengyang 233100, China

**Keywords:** bile acids, 16S rRNA sequencing, intestinal microbiota, fatty liver, laying hens

## Abstract

**Simple Summary:**

Bile acids (BAs) are metabolic byproducts of cholesterol in liver cells and play an important role in fat metabolism. Intensive and high-density feeding practices can impose a heavy burden on animal livers, and even lead to liver diseases such as fatty liver, cirrhosis, and liver toxicity. Therefore, this study investigated the effect of bile acid supplementation in fatty liver hemorrhagic syndrome, production performance, and physiological and quality characteristics of laying hen eggs. In summary, BA supplementation significantly improves the production performance and egg quality in high-fat diet (HFD)-treated laying hens, by enhancing antioxidant capacity, modulating the inflammatory response, as well as altering the intestinal microbiota composition. These findings underscore the multifaceted benefits of BAs supplementation in poultry nutrition, offering a promising strategy to optimize egg production and hen health.

**Abstract:**

This study aimed to investigate the effects of bile acids (BAs) supplementation on fatty liver hemorrhagic syndrome (FLHS), production performance, and physiological and quality characteristics of laying hen eggs. Sixty Sanhuang laying hens, aged 28 weeks, were randomly allocated to six dietary treatments over a 4-week period, including the control (CON) group (feeding basal diet), the high-fat diet (HFD)-treated group (basal diet containing 10% soybean oil), and HFD supplemented with 0.01% and 0.02% of chenodeoxycholic acid (CDCA) or hyodeoxycholic acid (HDCA) groups. Production performance, egg quality, liver morphology, serum biochemical indexes, antioxidant capacity, proinflammatory cytokines, and intestinal microbiota were evaluated. The average body weight in 0.01% CDCA was larger than in the HFD group (*p* < 0.05). Eggshell Thickness in the CON group was greater than in the HFD, 0.01% CDCA, and HDCA groups (*p* < 0.05). Albumen height in the 0.02% HDCA group was higher than the HFD group (*p* < 0.05). Eggshell weight in the HFD group was less than the CON group (*p* < 0.05). Haugh unit (HU) in the HDCA group was larger than the HFD group (*p* < 0.05). Albumen weight in the 0.02% HDCA group was greater than the CON and HFD groups (*p* < 0.05). In the HFD group, the levels of triglyceride (TG), total cholesterol (TC), and low-density lipo-protein cholesterol (LDL-C) were surpassing the other groups (*p* < 0.05). The levels of catalase (CAT) and total superoxide dismutase (T-SOD) in the HFD group was smaller than the other groups (*p* < 0.05). The level of malondialdehyde (MDA) in the HFD group was higher than in the other groups (*p* < 0.05). Tumor necrosis factor-α (TNF-α) levels were larger in the HFD group than in the other groups (*p* < 0.05). The 16S rRNA sequencing analysis indicated significant variations in the relative abundance of specific bacterial populations among the different treatment groups. The treatment and CON groups exhibited a higher presence of bacteria that inhibit host energy absorption or promote intestinal health such as *Firmicutes*, *Bacteroidetes*, and *Ruminococcus*, whereas the HFD group showed an increased prevalence of potentially pathogenic or deleterious bacteria, such as *Desulfovibrio* spp. In conclusion, the supplementation of BAs in poultry feed has been demonstrated to effectively mitigate the detrimental effects of FLHS in laying hens. This intervention regulates lipid metabolism, bolsters antioxidant defenses, reduces inflammation, and modulates the gut microbiota, offering a novel perspective on the application of BAs in the poultry industry.

## 1. Introduction

The causes, mechanisms, and pathological symptoms of chicken fatty liver hemorrhagic syndrome (FLHS) are comparable to those of mammalian non-alcoholic fatty liver disease (NAFLD); therefore it is frequently employed as an animal model for NAFLD-related research [1]. Affected laying hens may experience severe suffering as a result of FLHS. Their capacity to act normally is compromised by the physical discomfort brought on by the buildup of fat in the liver [2]. The etiology of FLHS is multifactorial, involving dysregulation in lipid metabolism influenced by various factors such as nutritional intake, metabolic processes, environmental factors, hormonal imbalances, and genetic predisposition [3]. Among these factors, nutrition has been identified as the primary determinant of FLHS [4]. Recent research has demonstrated the ability of dietary additives and a balanced diet structure to mitigate flora abnormalities and slow the progression of liver disease [5]. Diets high in fat change the ecosystem of gut bacteria, which can lead to serious liver disorders [6]. The widespread use of cages in the laying hens rearing industry, along with limited exercise, increased production demands, and fluctuating temperatures, disrupts hepatic lipid metabolism, rendering hens more susceptible to FLHS, a condition that significantly impacts the poultry industry, and 74% of necropsied hens die due to FLHS [7,8].

Bile acids (BAs) constitute the key component of bile and are generated by the liver from cholesterol [9]. Conjugated bile acids and free bile acids are the two types of amphiphilic steroid molecules that are created when hepatocytes break down cholesterol [10]. Secondary metabolites are formed through 7α-dehydroxylation or epimerization processes, such as cholic acid (CA) to deoxy-cholic acid (DCA) and ursodeoxycholic acid (UDCA) to lithocholic acid (LCA). Bitter acids like alpha-bitter acid (α-MCA) and beta-bitter acid (β-MCA) undergo metabolic transformations into ω-bitter acid (ω-MCA), hydroxydeoxycholic acid (HDCA), and hydroxycholic acid (HCA), etc [11]. Animal bile has been used as a traditional Chinese medicine for thousands of years in China [12], among which porcine bile is more widely used due to its rich source and low price [13]. The main components of porcine bile include chenodeoxycholic acid (CDCA) and HDCA. CDCA has established applications in treating gallstones and other liver diseases [14]. It has been demonstrated that CDCA possesses anti-inflammatory, hypertensive, decongestant, asthma, and immunosuppressive pharmacological activities [15]. HDCA has antispasmodic, hypolipidemic, and expectorant properties; it is used clinically in situations of hyperlipidemia [16]. In previous studies, it was discovered that alterations in the abundance of specific genera, such as *Bacteroides* and *Lachnospiraceae*, are closely associated with the extent of liver fibrosis and the severity of nonalcoholic steatohepatitis in hens [17]. BAs play an important role in controlling the expression of hepatic lipogenic genes and enhancing intestinal lipase activity [18]. Moreover, the existing studies on laying hens suggested that a high-fat diet (HFD) can lead to nonalcoholic steatohepatitis and dysbiosis of the intestinal microbiota [19]. However, the underlying mechanisms remain to be fully understood. Therefore, we hypothesized that supplementation with BAs could ameliorate lipid accumulation and intestinal flora dysbiosis in the FLHS liver. In the present study, we explored the effects of BA supplementation on FLHS, production performance, and physiological and quality characteristics of laying hen eggs, in order to provide a theoretical basis for the treatment of fatty liver in animals.

## 2. Materials and Methods

### 2.1. Animals and Treatments

At 28 weeks of age, sixty Sanhuang hens showing no clinical signs of disease, having normal body temperature, regular fecal output, and appropriate feathering were randomly assigned to six dietary treatments. Each group has 10 chickens [20], each chicken is kept in a single cage; the cage height is 400 mm, width is 250 mm, and length is 330 mm. The control group (CON) was fed a basal diet, while the other groups received supplemented HFD (containing 10% soybean oil), and BA treatment groups were supplemented with different concentrations of CDCA (0.01% and 0.02%) or HDCA (0.01% and 0.02%) for 4 weeks. Hens were allowed ad libitum access to experimental diets and water. The sheds also had controlled ventilation and lighting, with a schedule of 16 h of light followed by 8 h of darkness (16L:8D). Using maize and soybean meal, the basal diet was created in accordance with the Agricultural Trade Standardization of China’s nutrient levels and composition (NY/T33-2004) (Table 1). The BAs were obtained from Anhui Chem-bright Bioengineering Co., Ltd. (Huaibei, China), with a purity of approximately 99.9%.

### 2.2. Production Performance

The quantity and weight of eggs were recorded daily. Daily feed consumption was also recorded, along with daily mortality rates throughout the entire experiment. For the overall experimental period, key performance indicators, including the laying rate (LR), egg mass, average egg weight (AEW), body weight, average daily feed intake (ADFI), and feed conversion ratio (FCR), were calculated as follows:

ADFI = Total feed intake/Day
AEW = Total egg weight/number
FCR = ADFI/AEW
LR = Number of eggs laid/number of days to lay eggs × 100%

### 2.3. Egg Quality Traits Evaluation

A total of 222 eggs were collected for egg quality measurements over a 5-day period from day 24 to day 28 of the experiment. Initially, the eggs were weighed using an electronic scale (Mettler-Toledo International Inc, Shanghai, China). The longitudinal and transverse diameters of the eggs were measured with a vernier caliper (Yantai Lvlin Tools Co., Ltd., Yantai, China). The egg shape index was calculated by dividing the longitudinal diameter by the transverse diameter. The egg-specific gravity was determined using the brine-floating method. Eggshell strength or resistance to breakage was assessed using an analog eggshell strength meter (RunHu Instruments Co., Ltd., Guangzhou, China). Eggshell thickness was measured at three distinct segments—the top, middle, and bottom—using a vernier caliper, and the mean value of these measurements was taken as the representative overall eggshell thickness (Yantai Lvlin Tools Co., Ltd., Yantai, China). Eggs were transferred onto a clean and flat surface and albumen height was measured using a vernier caliper and used to calculate the Haugh unit (HU). Albumen height was the value (mm) obtained when the tip of a vernier caliper touched the egg white at a distance of 1 cm around the yolk. Then, yolk and albumen were separated, and yolk weight and albumen weight were measured using an electronic scale (Mettler-Toledo International Inc, Shanghai, China). After determining the albumen height, the HU was calculated based on the corresponding egg weight using the equations described below:

HU = 100 log (H − 1.7W^0.37^ + 7.57)
H: albumen height (mm)
W: egg weight (g)

### 2.4. Liver Morphology

The Random Number Table method was employed for sampling procedures, resulting in the collection of three samples from each group for analysis. The hens corresponding to these randomly generated numbers were euthanized by dislocation, after which liver tissue samples were collected from three different regions of each hen’s liver, and 1.5 × 1.5 × 0.2 cm^3^ sections were excised and preserved in 10% neutral buffered formalin. After the fixing solution was removed, all liver specimens were processed using conventional paraffin embedding techniques. Each sample was then sectioned into 5-µm slices and mounted on glass slides. Hematoxylin-eosin (H&E) staining was performed for examination under light microscopy (Motic Group Co., Ltd., Guangzhou, China). For each section, at 200×, 10 different fields of view were selected and scored individually. The histological lesions were staged, according to a scoring system, into four categories: 0 = no abnormalities; 1 = moderate fatty liver (only cells from one zone are affected; periportal, midzonar, or zentrolobular); 2 = severe fatty liver (the periportal and midzonal or midzonal to centrolobular areas are affected); 3 = very severe fatty liver (all three zones are affected, including the Kupffer cells) [21]. The mean was calculated as the score for that slice, and then the means ± SEM of the three slices were counted and statistically analyzed.

### 2.5. Serum Biochemical Indexes

After the trial ended, a 5 mL blood sample was collected from the vein beneath the wing following an overnight fast. The samples were then allowed to stand at room temperature for 2 h before being centrifuged at 1000× *g* for 10 min. After centrifugation, the serum was extracted and stored at −80 °C for determination of serum biochemical parameters. Serum biochemical indicators including total aspartate transaminase (AST, C010-1), alanine aminotransferase (ALT, C009-1-1), triglyceride (TG, A110-1-1), total cholesterol (TC, A111-1-1), high-density lipoprotein cholesterol (HDL-C, A112-1-1), and low-density lipo-protein cholesterol (LDL-C, A113-1-1) were purchased from Nanjing Jiancheng Bioengineering Research Institute Co., Ltd. (Nanjing, China), and the assays were performed in accordance with the instructions. The levels of these biochemical indexes are closely associated with the occurrence of fatty liver, and changes in these parameters can help assess the degree of liver damage, monitor the progression of the disease, and guide treatment plans [22].

### 2.6. Antioxidant Capacity

Glutathione catalase (GSH-Px, A005-1-2), total superoxide dismutase (T-SOD, A001-1-1), total antioxidant capacity (T-AOC, A015-3-1), malondialdehyde (MDA, A003-2), glutathione (GSH, A006-2-1) and catalase (CAT, A007-1-1) play crucial roles in regulating oxidative stress. Serum antioxidant levels were detected using kits from Nanjing Jiancheng Bioengineering Institute (Nanjing, China), following the manufacturer’s instructions.

### 2.7. ELISA Assay

Interleukin-6 (IL-6, I19028664), IL-1β (I06028611), and tumor necrosis factor-α (TNF-α, I05028610) act as pro-inflammatory factors that promote inflammatory responses and affect adipocyte metabolism and differentiation. In the experiment, the serum was collected from the laying hens and analyzed using ELISA kits (Cusabio, Wuhan, China), and the assays were performed following the manufacturer’s instructions.

### 2.8. 16S rRNA Gene Sequencing

Cecal contents from laying hens were collected and stored at −80 °C. The Magnetic Soil and Stool DNA Kit was obtained from Tiangen Biotech Co. (Beijing, China) to extract total DNA from the cecal contents of the hens. Specific operations were performed according to the instructions. The library was constructed using the NEB Next^®^ Ultra DNA Library Prep Kit (San Diego, CA, USA), and its integrity was confirmed using an Agilent 5400 system (Agilent Technologies Co., Ltd., San Diego, CA, USA), and quantified by qPCR. After the library was qualified, it was sequenced on the Illumina sequencing platform.

### 2.9. Bioinformatics Analysis

Using the QIIME2 tools import application, raw data FASTQ files were imported into a format that the QIIME2 system could use. To create the feature table of amplicon sequence variant (ASV), demultiplexed sequences from each sample were quality filtered and trimmed, de-noised, and merged, and then the chimeric sequences were detected and deleted using the QIIME2 dada2 plugin [23]. To create the taxonomy table, ASV sequences were aligned to a pre-trained GREENGENES 13_8 99% database (trimmed to the V3V4 region bound by the 338F/806R primer pair) using the QIIME2 feature-classifier plugin [24]. To remove any potentially contaminated mitochondrial and chloroplast sequences, the QIIME2 feature-table plugin was utilized.

In order to ensure the accuracy of Operational Taxonomic Units (OTUs) clustering and subsequent analyses, the raw sequencing data will first be filtered and processed, and the processed data will be filtered to obtain valid data. Then, based on the valid data, OTUs clustering/de-noising and species classification analyses were performed to form species abundance profiles of OTUs and other species taxonomic classes [25]. QIIME2 diversity plugin was used to analyze Alpha diversity. The Alpha Diversity Index is an analysis of the diversity of species in a sample that includes both richness and evenness of the species composition of the sample and is usually assessed using indices such as Faith’s phylogenetic diversity (Faith-pd), Observed features, and Shannon curves. The higher the index, the more complex the diversity of a sample. The R language Phyloseq package visualized the results and assessed differences in species complexity between samples using β-diversity analysis. Multiple comparisons were performed between subgroups using the DESeq2 method to find microorganisms that differed significantly between every two subgroups [26]. Correlation heatmaps were drawn using mainly the R language pheatmap package. Correlation heatmaps can be used to analyze whether there is a significant correlation between environmental factors or other clinical phenotypic data and microbial communities or species, and then calculate Spearman’s correlation coefficients between the environmental factors and the microbial species and present them as heatmaps.

### 2.10. Statistical Analysis

Statistical analysis was performed using SPSS 23.0 software, employing one-way analysis of variance (ANOVA). Differences between treatments were further assessed using Duncan’s range of comparison tests. The experimental data were expressed as the means ± SEM, and *p* < 0.05 between groups was considered as statistically significant differences. Microbial diversity analysis was performed using the Guangzhou Wekemo Bioincloud platform (https://www.bioincloud.tech/ (accessed on 15 December 2023)).

## 3. Results

### 3.1. Production Performance

As shown in Table 2, the average body weight in 0.01% CDCA was larger than the HFD group (*p* < 0.05). LR in 0.02% CDCA was less than treatment groups (*p* < 0.05). However, the supplementation of CDCA and HDCA in the diet did not show any significant effects (*p* > 0.05) on the ADFI, Egg mass, AEW, and FCR of laying hens.

### 3.2. Egg Quality Traits Evaluation

As shown in Table 3, the addition of CDCA and HDCA to the diet had no significant (*p* > 0.05) effect on the eggshell strength, yolk weight, egg weight, and egg shape index in laying hens. Eggshell Thickness in the CON group was greater than in the HFD, 0.01% CDCA, and HDCA groups (*p* < 0.05). Albumen height in the 0.02% HDCA group was higher than in the HFD group (*p* < 0.05). Egg shell weight in the HFD group was less than the CON group (*p* < 0.05). Haugh unit in the HDCA group was larger than in the HFD group (*p* < 0.05). Specific gravity of egg in the 0.01% CDCA group was smaller than in the CON group (*p* < 0.05). Albumen weight in the 0.02%HDCA group was greater than the CON and HFD groups (*p* < 0.05).

### 3.3. Liver Morphology

**Figure 1:**
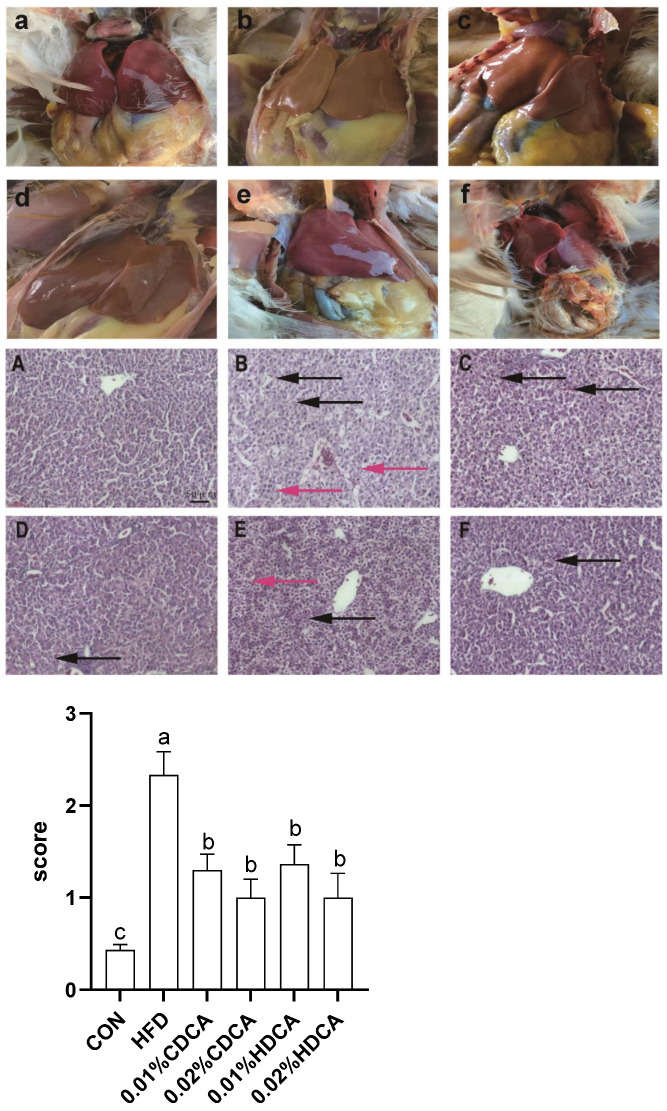
Effects of bile acids on pathological changes and lipid accumulation in liver tissue. Images of (**A**,**a**) normal liver tissue, (**B**,**b**) HFD, (**C**,**c**) 0.01% CDCA, (**D**,**d**) 0.02% CDCA, (**E**,**e**) 0.01% HDCA and (**F**,**f**) 0.02% HDCA. Red arrow indicates lipid droplets and fat vacuoles. Black arrow indicates amid intraparenchymal hemorrhage. The values of different lowercase letters were significantly different (*p* < 0.05).

The macroscopic liver appearance of layers from the HFD group showed obvious grayish-yellow, hepatomegaly, and brittleness compared with the appearance of layers from the control group (Figure 1a–f). Meanwhile, the accumulation of lipid droplets and fat vacuoles in the liver sections of the laying hens in the HFD group averaged 2.33 ± 0.15. A small number of fat vacuoles were seen in the treatment groups, for the 0.01% CDCA was 1.30 ± 0.1, the 0.02% CDCA was 1.00 ± 0.12, the 0.01% HDCA was 1.37 ± 0.12, and the 0.02% HDCA was 1.00 ± 0.15. The hepatocyte structure was clear and without fat vacuoles in the liver section of layers from the control group, with an average of 0.43 ± 0.03 (Figure 1A–F). 

### 3.4. Serum Biochemical Indexes

The effect of dietary BAs on serum biochemical indexes is presented in Table 4. In the HFD group, the levels of TG, TC, and LDL-C were surpassing the other groups (*p* < 0.05). In the HFD and 0.01% CDCA groups, the levels of ALT were larger than the CON, 0.02% CDCA, and 0.02% HDCA groups (*p* < 0.05).

### 3.5. Antioxidant Capacity

Antioxidant activity indicators are listed in Table 5. The levels of CAT and T-SOD in the HFD group were smaller than the other groups (*p* < 0.05). The level of MDA in the HFD group was higher than the other groups (*p* < 0.05). In the HFD group, the level of T-AOC was less than the CON, 0.01% CDCA, and HDCA groups (*p* < 0.05). GSH-PX serum levels were higher in the CON and 0.02% CDCA groups than in the HFD group (*p* < 0.05).

### 3.6. Proinflammatory Cytokines

As shown in Table 6, the levels of serum IL-6 were higher in the HFD group than in CON and 0.02% HDCA (*p* < 0.05). The level of IL-1β in CON, 0.02% CDCA, and 0.02% HDCA groups was lower than the HFD group (*p* < 0.05). TNF-α levels were larger in the HFD group than in the other groups (*p* < 0.05). 

### 3.7. Correlation Analysis

#### 3.7.1. Abundance

**Figure 2:**
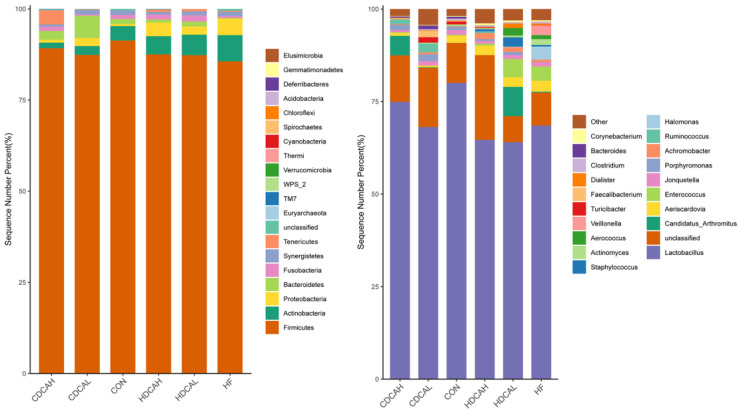
The relative abundance of the intestinal microbiota community structure. Relative abundance of intestinal microbiota at the phylum level (**left**). Relative abundance of intestinal microbiota at the genus level (**right**). CON: normal liver tissue. HF: high fat. CDCAL: 0.01% CDCA. CDCAH: 0.02% CDCA. HDCAL: 0.01% HDCA. HDCAH: 0.02% HDCA.

The relative abundance was invested at the phylum and genus levels (Figure 2). At the phylum level, *Firmicutes*, *Actinobacteria*, *Proteobacteria*, and *Bacteroidetes* were the most abundant phyla in the cecum of laying hens, together comprising over 90% of the entire cecal microbiome. In addition, the abundance of *Actinobacteria*/*Proteobacteria* in the HFD group was higher than in other groups, while that of *Firmicutes* and *Bacteroidetes* in the HFD group was lower than in other groups. At the genus level, *Lactobacillus* accounted for nearly 70% of the total cecum bacteria. The abundance of *Porphyromonas* decreased in the HFD group compared with other groups. It was noteworthy that the abundance of *Aeriscardovia*, *Halomonas* and *Actinomyces* increased in the HFD group compared to other groups.

#### 3.7.2. Alpha

**Figure 3:**
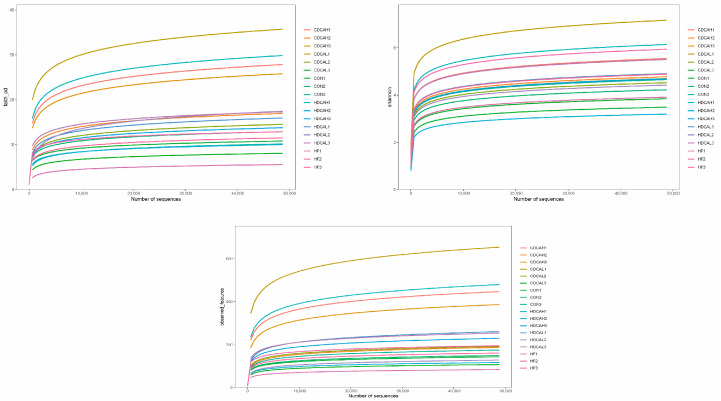
The alpha diversity was assessed using the Faith-pd, Observe-features, Shannon. CON: normal liver tissue. HF: high fat. CDCAL: 0.01% CDCA. CDCAH: 0.02% CDCA. HDCAL: 0.01% HDCA. HDCAH: 0.02% HDCA.

Faith’s phylogenetic diversity (Faith−pd), Observed features, and Shannon curves were meticulously crafted to encapsulate the intricacies of microbial diversity across varying sequencing depths of each sample. As delineated in Figure 3, a discernible upsurge in the species richness of the chicken’s gut microbiota was observed with an escalating number of sequences, until a certain point where the curve began to plateau, signifying that the sequencing data had amassed to a volume sufficient to capture the bulk of microbial information present in the samples.

#### 3.7.3. Beta

**Figure 4:**
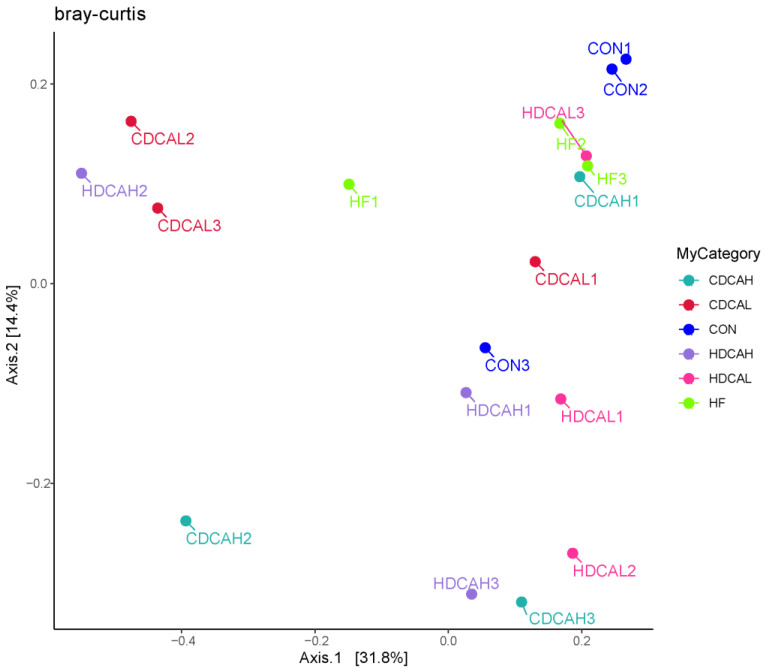
PCoA based on Bray−Curtis theory. CON: normal liver tissue. HF: high fat. CDCAL: 0.01% CDCA. CDCAH: 0.02% CDCA. HDCAL: 0.01% HDCA. HDCAH: 0.02% HDCA.

In order to determine how similar sample groups are to one another, beta diversity primarily explains the fluctuation of species composition on spatial scales. Principal Coordinate Analysis (PCA), which can display the similarities and differences between groups, can be used to perform beta diversity analysis. The closer a species composition is to the graph, the more similar it is, and the less similar it is, the farther it is from the coordinate system. Bray Curtis distance is the most commonly used indicator of the difference between response communities in ecology. PCoA analysis was performed based on Bray Curtis distance, and the principal coordinate combination with the largest contribution rate was selected for mapping, as seen in Figure 4, the degree of dispersion between individuals in the CDCA and HDCA groups was more pronounced than that in the CON and HFD, indicating that there was a greater degree of individual variation in the structure of the intestinal flora of the test groups. The explanation rate of PCo1 was 31.8%, and that of PCo2 was 14.4%. The aforementioned findings suggest that the intestinal flora structure of laying hens across various groups can be more accurately reflected by P-CoA analysis.

#### 3.7.4. Variance Analysis

**Figure 5:**
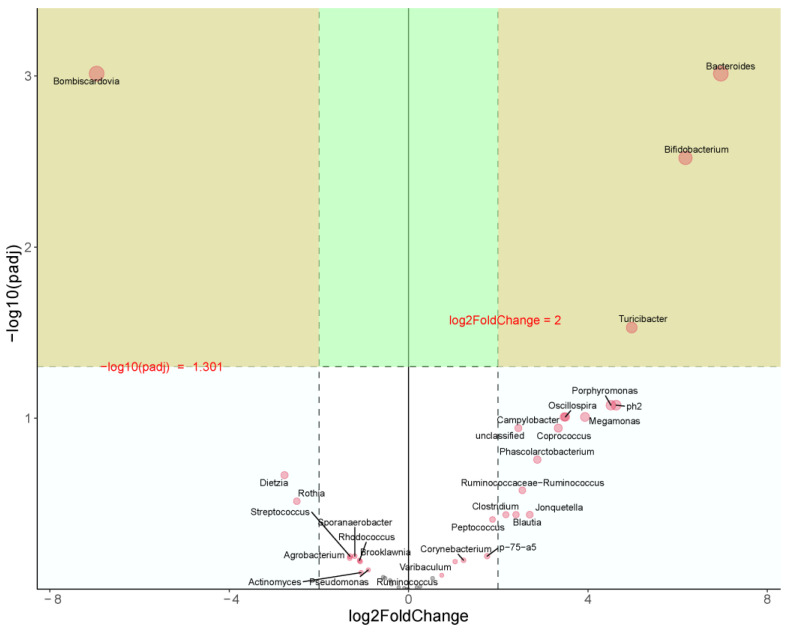
Volcano plot of differential bacteria. CON vs. HFD: log2 FoldChange > 2, *p* < 0.05.

As shown in Figure 5, at the genus level, CON vs. HFD each point represented a species attribute, log2FoldChange > 2, *p* < 0.05. Compared with the CON group, *Bacteroides*, *Bifidobacterium*, and *Turicibacter* were significantly up-regulated while *Bombiscardovia* was significantly down-regulated in the HFD group.

#### 3.7.5. Correlation Analysis between Production Performance and Intestinal Microbiota

**Figure 6:**
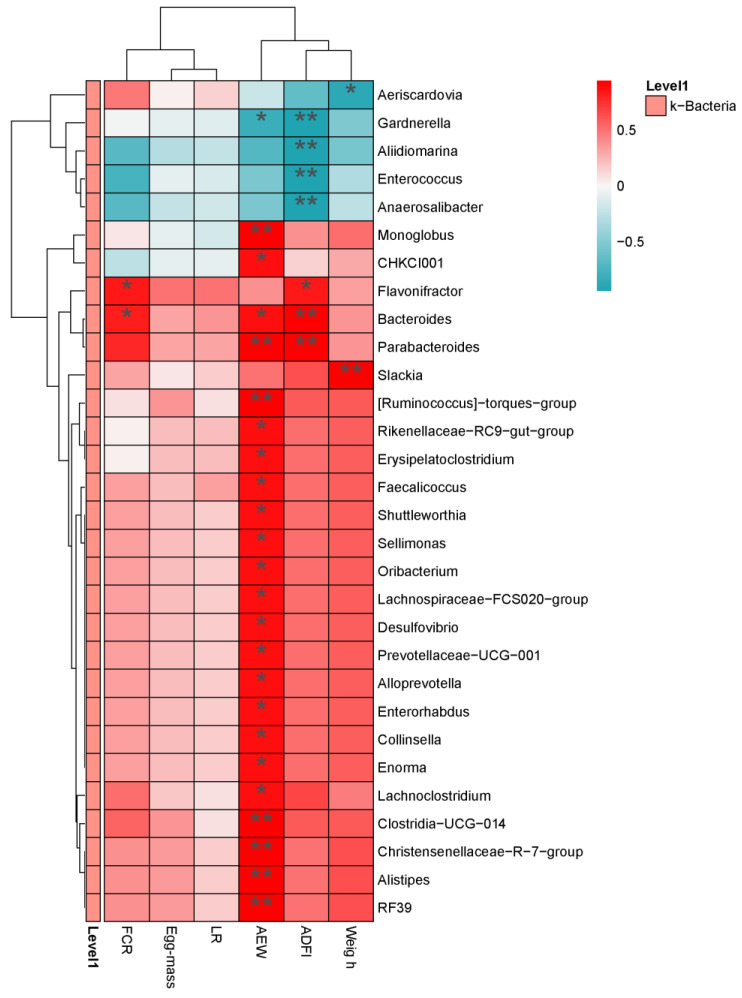
Correlation analysis between production performance and intestinal microbiota. Horizontal for production performance, Vertical for the genus differential bacteria. Red for positive correlation, green for negative correlation. * *p* < 0.05, ** *p* < 0.01.

As shown in Figure 6, the species community heat maps reflect the composition of both dominant and subdominant bacterial species within each group. At the genus level, *Flavonifractor* and *Bacteroides* were positively correlated with FCR (*p* < 0.05). CHKCI001, *Bacteroides*, Rikenellaceae_RC9_gut_group, *Erysipelatoclostridium*, *Faecalicoccus*, *Shuttleworthia*, *Sellimonas*, *Oribacterium*, *Desulfovibrio* spp, *Prevotellaceae*, *Allo-prevotella*, *Enterorhabdus*, *Collinsella*, *Enorma*, and *Lachnoclostridium* were positively correlated with AEW (*p* < 0.05), *Monoglobus*, *Parabacteroides*, *Alistipes*, and RF39 were positively correlated with AEW (*p* < 0.01), and *Gardnerella* was negatively correlated with AEW (*p* < 0.05). *Gardnerella*, *Aliidiomarina*, *Enterococcus*, and *Anaerosalibacter* were negatively correlated with ADFI (*p* < 0.01), *Flavonifractor* was positively correlated with ADFI (*p* < 0.05), and *Bacteroides* and *Parabacteroides* were positively correlated with ADFI (*p* < 0.01). *Slackia* was highly significant and positively correlated with weight (*p* < 0.01), *Aeriscardovia* was negatively correlated with weight (*p* < 0.05).

#### 3.7.6. Correlation Analysis between Egg Quality and Intestinal Microbiota

**Figure 7:**
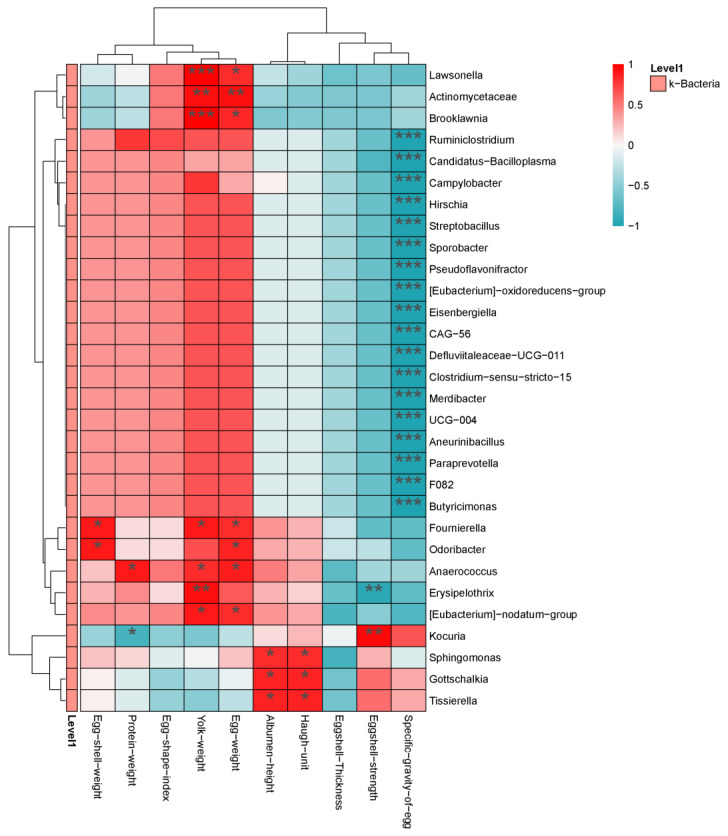
Correlation analysis between egg quality and intestinal microbiota. Horizontal for egg quality, Vertical for the genus differential bacteria. Red for positive correlation, green for negative correlation. * *p* < 0.05, ** *p* < 0.01, *** *p* < 0.001.

The correlation between intestinal flora and egg quality was revealed by correlation analysis (Figure 7). At the genus level, *Fournierella* and *Odoribacter* were positively correlated with eggshell weight (*p* < 0.05), and *Anaerococcus* was positively correlated with albumen weight (*p* < 0.05). *Kocuria* was negatively correlated with albumen weight (*p* < 0.05). *Lawsonella*, *Actinomycetaceae*, *Brooklawnia*, *Fournierella*, *Odoribacter*, *Anaerococcus*, and *Erysipelothrix* were positively correlated with yolk weight and egg weight (*p* < 0.05). *Sphingomonas*, *Gottschalkia*, and *Tissierella* were positively correlated with HU and Albumen height (*p* < 0.05). *Erysipelothrix* was negatively correlated with Eggshell strength (*p* < 0.01), and *Kocuria* was positively correlated with Eggshell strength (*p* < 0.01). Egg−specific gravity was negatively correlated with most intestinal microbiota.

#### 3.7.7. Correlation Analysis between Intestinal Microbiota and Serum Biochemical Indexes

**Figure 8:**
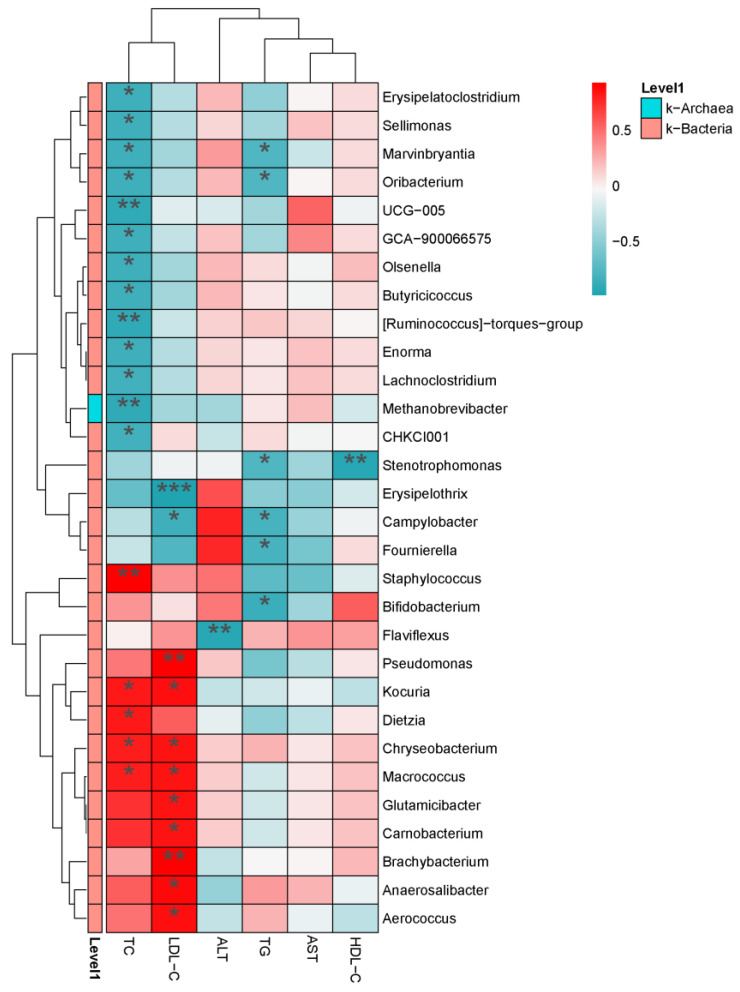
Correlation analysis between intestinal microbiota and serum biochemical indexes. Horizontal for serum biochemical indexes, Vertical for the genus differential bacteria. Red for positive correlation, green for negative correlation. * *p* < 0.05, ** *p* < 0.01, *** *p* < 0.001.

BAs can not only alter the composition of intestinal microbiota but also improve the biochemical indices in the serum of HFD-treated laying hens. Therefore, the correlation between intestinal microbiota and serum biochemical indexes was revealed by Spearman correlation analysis (Figure 8). At the genus level, *Erysipelatoclostridium*, *Sellimonas*, *Marvinbryantia*, *Oribacterium*, *UCG-005*, *GCA-900066575*, *Olsenella*, *Butyricicoccus*, *Enorma*, *Lachnoclostridium*, *Methanobrevibacter*, and *CHKCI001* were negatively correlated with TC (*p* < 0.05), while *Staphylococcus*, *Kocuria*, *Dietzia*, *Chryseobacterium*, and *Macrococcus* were positively correlated with TC (*p* < 0.05). *Pseudomonas*, *Kocuria*, *Chryseobacterium*, *Macrococcus*, *Glutamicibacter*, *Carnobacterium*, *Brachybacterium*, *Anaerosalibacter*, and *Aerococcus* were positively correlated with LDL-C (*p* < 0.05); nevertheless, *Erysipelothrix* and *Campylobacter* were negatively correlated with LDL-C (*p* < 0.05). *Flaviflexus* was negatively correlated with ALT (*p* < 0.01). *Marvinbryantia*, *Oribacterium*, *Stenotrophomonas*, *Campylobacter*, *Fournierella*, and *Bifidobacterium* were negatively correlated with TG (*p* < 0.05). *Stenotrophomonas* was negatively correlated with HDL-C (*p* < 0.01). These results suggested that the improvement of biochemical parameters in HFD-treated laying hens before and after administration was associated with intestinal microbiota.

#### 3.7.8. Correlation Analysis between Intestinal Microbiota and Antioxidant Capacity

**Figure 9:**
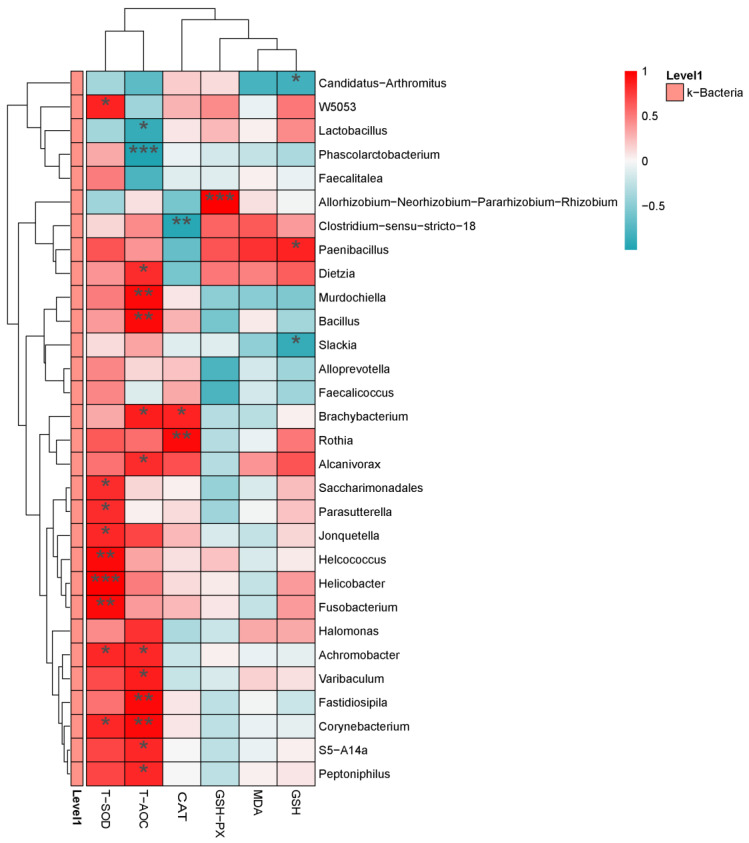
Correlation analysis between intestinal microbiota and antioxidant capacity. Horizontal for antioxidant capacity, Vertical for the genus differential bacteria. Red for positive correlation, green for negative correlation. * *p* < 0.05, ** *p* < 0.01, *** *p* < 0.001.

A heat map was used to exhibit the associations between intestinal microbiota and antioxidant capacity (Figure 9). At the genus level, T-SOD and T-AOC were positively correlated with most gut microbiota. *Clostridium-sensu-stricto-18* was negatively correlated with CAT (*p* < 0.01), *Brachybacterium* and *Rothia* were positively correlated with CAT (*p* < 0.05). *Allorhizobium–Neorhizobium–Pararhizobium–Rhizobium* was positively correlated with GSH-PX (*p* < 0.001), *Paenibacillus* was positively correlated with GSH (*p* < 0.05), and *Candidatus*-*Arthromitus* and *Slackia* were negatively correlated with GSH (*p* < 0.05).

#### 3.7.9. Correlation Analysis between Intestinal Microbiota and Proinflammatory Cytokines

**Figure 10:**
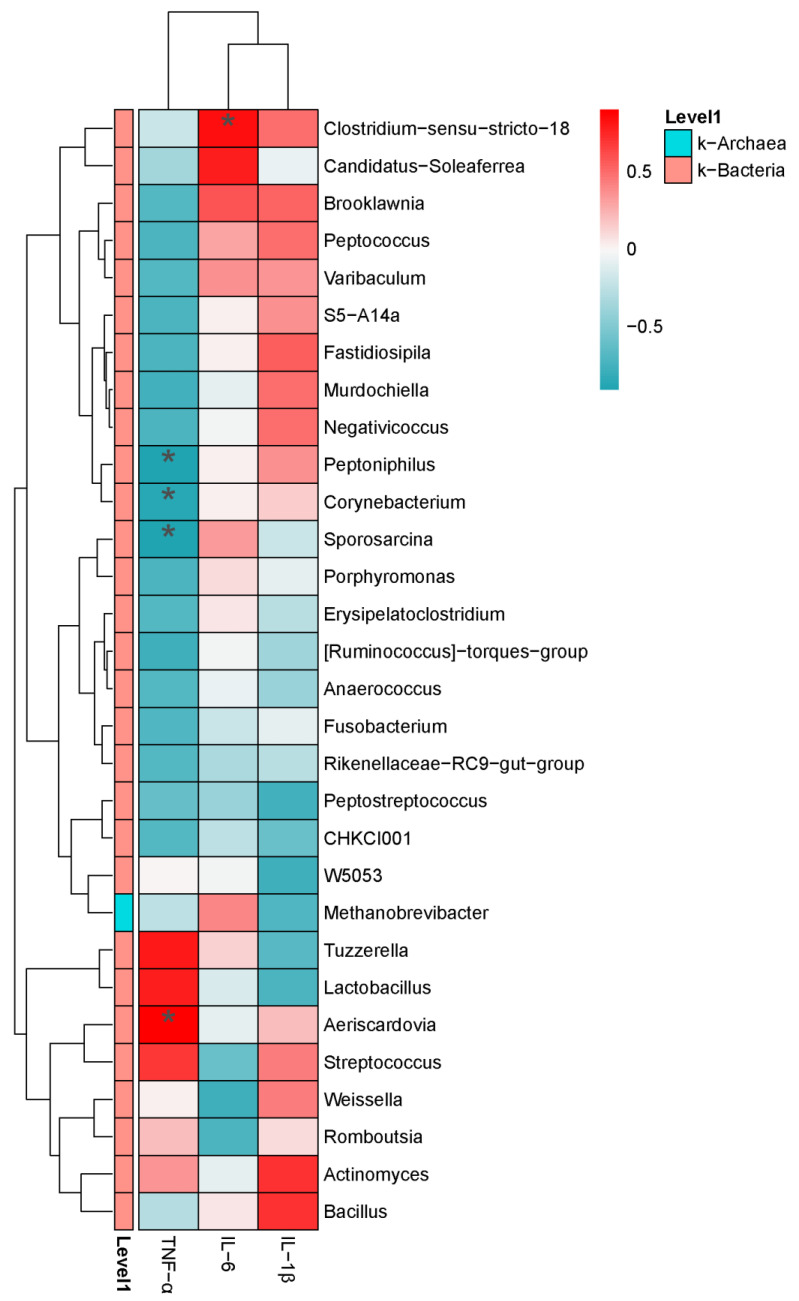
Correlation analysis between intestinal microbiota and proinflammatory cytokines. Horizontal for proinflammatory cytokines, Vertical for the genus differential bacteria. Red for positive correlation, green for negative correlation. * *p* < 0.05.

As shown in Figure 10, *Peptoniphilus*, *Corynebacterium*, and *Sporosarcina* were negatively correlated with TNF-α (*p* < 0.05), while *Aeriscardovia* was positively correlated with TNF-α (*p* < 0.05). *Clostridium-sensu-stricto-18* was positively correlated with IL-6 (*p* < 0.05).

## 4. Discussion

The use of 28-week-old hens in the study was dictated by the convenience of hen availability. Extensive research has demonstrated that BAs possess considerable therapeutic potential for the prevention of lipid metabolism disorders [27,28]. However, the effect of CDCA and HDCA on FLHS in laying hens remains unclear, necessitating further investigation to elucidate their impact [29]. In the present study, modified from previous studies, HFD was used to induce FLHS in laying hens [30]. After four weeks, the LR in 0.02% CDCA was less than treatment groups. Furthermore, Neijat et al. found that excessive fat consumption resulted in an increased metabolic burden and liver fat accumulation, which subsequently reduced the LR [31]. Excessive fat consumption can negatively impact the hens’ reproductive function, including their laying rate [21]. The body weight in the 0.01% CDCA group was more than the HFD group. Gi Ppeum Han et al. found that hens with higher BW generally exhibit higher egg weight supporting the increased egg weight with increased BW observed in the current study [32].

In the context of egg quality assessment, it is imperative to delve into the multifaceted aspects that contribute to its overall value. According to Young, eggshell strength, eggshell thickness, albumen height, HU, yolk percentage, and eggshell percentage did not differ significantly between the treatment groups over the 28-day and 56-day periods. On the other hand, yolk color dropped in both 28 and 56 days (quadratic and linear) with a dose of 3000 mg/kg of BAs, while other doses of BAs had no effect on yolk color [33]. In the present study, the HFD or BAs supplementation did not have any impact on eggshell strength, yolk weight, egg weight, or egg shape index, whereas albumen height, albumen weight, and HU were higher in the 0.02% HDCA group than in the HFD group. However, the CDCA and 0.01% HDCA had little effect on egg quality. Notably, the increase in egg quality in the 0.02% HDCA groups needs further investigation.

The underlying cause of FLHS is a disturbance of lipid metabolism, which typically shows up as hemorrhage and hepatic steatosis [34]. Changes in the activity of ALT and AST, two markers of liver function, can be used to estimate the degree of liver damage [35]. Elevated liver enzymes could be an indicator of liver injury, and liver injury is a key component of FLHS [30]. According to the findings of this study, serum ALT levels were relatively higher in the HFD group, indicating liver damage in laying hens. 0.02% CDCA or HDCA could significantly alleviate HFD-induced liver injury in laying hens. HDL-C helps the body eliminate cholesterol from the bloodstream, whereas LDL-C is in charge of transporting cholesterol [36]. The present results showed that LDL-C was higher in the HFD group, indicating increased fat content in the liver [37]. In clinical practice, TC and TG are the main indicators for monitoring blood lipid levels and are involved in interfering with fatty acid metabolism. Yang found that significant enhancements in the content of TG, LDL-C, and TBA in the liver were observed in the HFD group [38]. This trial showed that TG and TC were higher in the HFD group. From the results, it is clear that the addition of high concentrations of oils exacerbates the damage to the liver. The addition of different types and doses of BAs to feeds can alleviate liver damage caused by fats and oils.

Oxidative stress is caused by an increase in energy content in the diet combined with a decrease in antioxidants. The oxidative stress, in turn, leads to damage in the coverings of liver cells. Consequently, this damage affects the liver receptors responsible for fat transportation to the ovary, causing the accumulation of fats in the liver [39]. Ultimately, this chain of events culminates in the phenomenon known as fatty liver and oxidative stress-induced hepatotoxicity [40]. The antioxidant capacity of the body is reflected in T-AOC [41]. MDA, an end-product of lipid oxidation, plays a pivotal role in the process of lipid peroxidation, serving as a key indicator of oxidative stress within biological systems [42]. SOD and GSH-Px constitute the foremost line of enzymatic antioxidant defense mechanisms. These enzymes function as specialized scavengers of free radicals, effectively neutralizing them and thereby safeguarding cellular components from oxidative injury. Consequently, SOD and GSH-Px levels are considered reliable indicators of activated antioxidant enzyme systems within biological organisms [43]. According to the current study, BAs can improve the anti-oxidative stress ability of laying hens and increase CAT, GSH-Px, T-SOD, GSH and T-AOC levels in the serum. Furthermore, the supplementation of BAs resulted in a decrease in the amount of MDA in the blood, indicating a potential enhancement of the antioxidant ability of laying hens. However, the antioxidant properties of BAs in eggs need further studies.

Persistent activation of pro-inflammatory factors and inflammatory signaling pathways is important for the development of hepatic fibrosis and steatohepatitis [44]. Excess FFA directly stimulates the expression of TNF-α, and TNF-α and ROS enable further production and release of pro-inflammatory cytokines (IL-1β, IL-6, TNF-α) mainly through stimulation of the NF-κB and JNK1/2 inflammatory pathways [45]. In this study, proinflammatory cytokines such as IL-1β, IL-6, and TNF-α were upregulated in the HFD group and reversed in the therapy groups which was consistent with previous study [46]. These results suggested that BAs can improve the immunity of laying hens and reduce their inflammatory response.

BAs in the feed primarily target the intestine upon entering the body and are closely linked to other tissues through enterohepatic circulation. BAs primarily alter the composition to change the intestinal microbiota, which can occur at the phylum and genus levels. Munukka et al. investigated whether liver fat content was associated with body composition, adipose tissue inflammation (via microarrays), and specific gut microbes (via 16S rRNA hybridization and flow cytometry) [47]. Herbert Tilg et al. found that endotoxin and phenylacetic acid, two metabolites produced by gut bacteria, are linked to the degree of steatosis in female NAFLD patients. When fed to mice, these metabolites and the microbiota from NAFLD patients caused hepatic fat accumulation [48]. As intestinal bacteria play an important role in the development of NAFLD in humans and rodents, it is speculated that intestinal bacteria also contribute to the development of FLHS in laying hens. The essential role of *Firmicutes*, *Bacteroidetes*, and *Ruminococcus* in maintaining gut health has been well documented. Specifically, *Firmicutes* plays a key role in energy metabolism [49]. The *Bacteroidetes* are responsible for metabolizing BAs and transforming toxic compounds by secreting butyric acid [50]. *Ruminococcus* can hinder the growth of harmful microorganisms by producing volatile fatty acids and lactic acid, which reduce the pH in the gut [51]. At the phylum level, the ratio of *Bacteroidetes* to *Firmicutes* was significantly decreased in the treatment and CON groups than that in the HFD group, which is consistent with previous studies on fatty liver in animals such as humans and mice [52]. However, both *Firmicutes Lactobacillus* and *Bacteroidetes* are beneficial for gut health [53]; their abundance was significantly higher in the CON group than in the HFD group. The abundance of Proteo-bacteria phylum is considered a sign of intestinal microbiota dysbiosis and a key factor in determining the intestinal health of animals, with significantly higher abundance in the HFD group than in the CON group. At the genus level, the abundance of *Actinomycetes* was significantly lower in the treatment and CON groups than in the HFD group, which is consistent with previous study [54].

FLHS is a metabolic disease closely associated with daily diet, hormones, and egg production. *Erysipelococcus* has been identified as being linked to inflammation and lipid metabolism [55]. *Erysipelococcus* may trigger an inflammatory response that affects the expression of SREBPs, which in turn affects lipid metabolism [56]. *Desulfovibrio* spp., as a pathogenic bacteria, is closely related to the occurrence of obesity and hyperlipidemia [57]. *Desulfovibrio* spp. contributes to inflammation by producing inflammatory factors like LPS and can also impact lipid metabolism by altering the composition and function of the gut microbiome [58]. *Bacteroides* strain has also been proven to regulate the redox level in the intestinal tract, creating favorable conditions for the host [59]. Our results showed that *Bacteroides* levels are positively associated with improved production performance, suggesting that an increase in probiotic content may be linked to maintaining a healthy physiological state in the host organism. *Lachnospiraceae* has also been shown to play a beneficial role in reducing obesity and was found to be negatively correlated with TC levels, while it was positively correlated with AEW. We hypothesize that the observed increase in pro-inflammatory and lipid metabolism-related bacteria, coupled with a decrease in probiotic populations in the HFD group, led to a dysbiosis of the gut microbiota characterized by both structural and functional alterations. This dysbiosis likely contributed to the pathological metabolic changes observed in the HFD group, thereby explaining the deviations from normal clinical parameters associated with HFD consumption. 16S rRNA sequencing has limitations in predicting the function of intestinal microbiota. Further investigation using metagenomic sequencing, targeted metabolomics, and transcriptomics is necessary to understand the detailed regulation of BAs on intestinal microbiota and liver metabolism.

## 5. Conclusions

In summary, BA supplementation had an impact on the production performance and egg quality in HFD-treated laying hens, enhancing antioxidant capacity, modulating the inflammatory response, as well as altering the intestinal microbiota composition. These findings underscore the multifaceted benefits of BA supplementation in poultry nutrition, offering a promising strategy to optimize egg production and hen health.

## Figures and Tables

**Table 1:** Ingredients and chemical composition of the basal diet (dry matter basis).

Items	CON	HFD	HFD			
			0.01% CDCA	0.02% CDCA	0.01% HDCA	0.02% HDCA
Ingredients, %						
Corn	61.00	61.00	60.99	60.98	60.99	60.98
Soybean meal	21.00	21.00	21.00	21.00	21.00	21.00
Wheat bran	10.00	0.00	0.00	0.00	0.00	0.00
Limestone	3.00	3.00	3.00	3.00	3.00	3.00
CDCA	0.00	0.00	0.01	0.02	0.00	0.00
HDCA	0.00	0.00	0.00	0.00	0.01	0.02
Soybean oil	0.00	10.00	10.00	10.00	10.00	10.00
Premix ^(1)^	5.00	5.00	5.00	5.00	5.00	5.00
Total	100.00	100.00	100.00	100.00	100.00	100.00
Nutrient levels ^(2)^, %						
ME (MJ/kg)	10.91	13.73	13.73	13.73	13.73	13.73
CP	17.49	17.7	17.63	17.09	17.78	17.82
CF	3.10	11.42	11.57	11.06	11.95	11.67
ASH	8.74	6.67	6.86	6.82	6.82	6.81
Ca	3.33	3.60	3.58	3.22	3.79	3.81
P	0.37	0.35	0.36	0.32	0.34	0.34
Met	0.27	0.25	0.23	0.21	0.26	0.28
Lys	0.64	0.62	0.67	0.66	0.63	0.67

^(1)^ The premix provided the following per kg of diets: VA 7500 IU, VD_3_ 3000 IU, DL-α-tocopheryl acetate 17.5 mg, D-biotin 0.13 mg, folic acid 0.75 mg, calcium-D-pantothenate 7.75 mg, cyanocobalamin 0.005 mg, riboflavin 5.00 mg, thiamin 2.25 mg, Cu (as copper sulfate) 9.00 mg, Fe (as ferrous sulfate) 60 mg, Mn (as manganese sulfate) 55 mg, Zn (as zinc sulfate) 60 mg; ^(2)^ ME was a calculated value, while the others were measured values. ME: metabolic energy, CP: crude protein, CF: crude fat, Ca: calcium, P: phosphorus, Met: methionine, Lys: lysine.

**Table 2:** Effects of dietary CDCA and HDCA supplementation on performance of laying hens ^1^.

Items ^2^	CON	HFD	HFD				*p* Value
			0.01% CDCA	0.02% CDCA	0.01% HDCA	0.02% HDCA	
ADFI	106.93 ± 5.66	96.38 ± 7.08	115.35 ± 6.43	103.51 ± 5.64	102.32 ± 5.77	96.43 ± 8.40	0.323
(g/d)							
AEW	47.80 ± 0.79	47.71 ± 1.07	49.24 ± 0.88	50.78 ± 2.08	48.17 ± 1.20	49.04 ± 0.54	0.133
(g)							
FCR	2.25 ± 0.12	2.10 ± 0.13	2.36 ± 0.15	2.07 ± 0.14	2.17 ± 0.13	1.92 ± 0.16	0.315
Body Weight	1.71 ± 0.07 ^c^	1.75 ± 0.11 ^bc^	2.06 ± 0.09 ^a^	2.01 ± 0.11 ^ab^	1.79 ± 0.09 ^abc^	1.70 ± 0.11 ^c^	0.022
(kg)							
Egg mass	31.39 ± 2.52	29.85 ± 2.16	35.99 ± 1.41	28.90 ± 2.71	34.77 ± 2.20	32.62 ± 2.42	0.29
(g/hen/d)							
LR	0.66 ± 0.06 ^ab^	0.62 ± 0.04 ^ab^	0.73 ± 0.03 ^a^	0.53 ± 0.07 ^b^	0.72 ± 0.04 ^a^	0.70 ± 0.02 ^a^	0.029

^1^ Values without letters or with the same superscript in the same row are not significantly different (*p* > 0.05), and the superscript values of different lowercase letters were significantly different (*p* < 0.05). Data are presented as the means ± SEM; ^2^ ADFI = average daily feed intake; AEW = average egg weight; FCR = feed conversion ratio; LR = laying rate; CON: normal control, HFD: addition of 10% of soybean oil to the base diet; addition of 0.01% CDCA, 0.02% CDCA, 0.01% HDCA and 0.02% HDCA to the HFD, respectively.

**Table 3:** Effects of dietary CDCA and HDCA supplementation on egg quality of laying hens ^1^.

Items	CON	HFD	HFD				*p* Value
			0.01% CDCA	0.02% CDCA	0.01% HDCA	0.02% HDCA	
Eggshell strength	35.77 ± 1.78	32.34 ± 1.57	32.01 ± 1.83	34.06 ± 2.41	36.47 ± 1.40	34.41 ± 2.28	0.155
(N/cm^2^)							
Eggshell	0.41 ± 0.007 ^a^	0.37 ± 0.009 ^bc^	0.36 ± 0.007 ^bc^	0.39 ± 0.011 ^ab^	0.37 ± 0.005 ^bc^	0.35 ± 0.007 ^c^	0.001
Thickness (mm)							
Albumen height	5.10 ± 0.16 ^ab^	4.75 ± 0.12 ^b^	4.97 ± 0.12 ^ab^	4.89 ± 0.21 ^ab^	5.21 ± 0.15 ^ab^	5.32 ± 0.13 ^a^	0.001
(mm)							
Yolk weight	16.71 ± 0.27	16.16 ± 0.39	16.78 ± 0.25	16.23 ± 0.36	16.08 ± 0.33	16.24 ± 0.34	0.42
(g)							
Egg weight	48.31 ± 0.64	48.2 ± 0.76	49.70 ± 0.58	48.83 ± 1.60	47.71 ± 0.84	49.66 ± 0.51	0.276
(g)							
Egg shell weight	4.55 ± 0.06 ^a^	4.21 ± 0.08 ^b^	4.43 ± 0.04 ^ab^	4.23 ± 0.11 ^ab^	4.30 ± 0.09 ^ab^	4.4 ± 0.09 ^ab^	0.004
(g)							
Egg shape	1.31 ± 0.011	1.32 ± 0.017	1.32 ± 0.008	1.31 ± 0.013	1.31 ± 0.010	1.32 ± 0.011	0.696
index							
Haugh	73.86 ± 1.15 ^ab^	71.30 ± 0.99 ^b^	72.37 ± 0.95 ^ab^	72.14 ± 1.41 ^ab^	75.13 ± 1.03 ^a^	75.25 ± 0.97 ^a^	0.001
unit							
Specific gravity	1.0787 ± 0.001 ^a^	1.0759 ± 0.001 ^ab^	1.0745 ± 0.001 ^b^	1.0754 ± 0.001 ^ab^	1.0763 ± 0.001 ^ab^	1.0773 ± 0.001 ^ab^	0.009
of egg							
albumen weight (g)	24.98 ± 0.52 ^b^	24.27 ± 0.55 ^b^	25.87 ± 0.49 ^ab^	25.76 ± 0.82 ^ab^	25.08 ± 0.50 ^ab^	26.49 ± 0.34 ^a^	0.042

^1^ Values without letters or with the same superscript in the same row are not significantly different (*p* > 0.05), and the superscript values of different lowercase letters were significantly different (*p* < 0.05). Data are presented as the means ± SEM. CON: normal control, HFD: addition of 10% of soybean oil to the base diet; addition of 0.01% CDCA, 0.02% CDCA, 0.01% HDCA, and 0.02% HDCA to the HFD, respectively.

**Table 4:** Effects of dietary CDCA and HDCA supplementation on serum biochemical indexes of laying hens ^1^.

Items ^2^	CON	HFD	HFD				*p* Value
			0.01% CDCA	0.02% CDCA	0.01% HDCA	0.02% HDCA	
ALT	20.04 ± 3.75 ^b^	36.31 ± 3.57 ^a^	34.33 ± 0.53 ^a^	17.70 ± 3.49 ^b^	24.54 ± 4.78 ^ab^	18.04 ± 2.82 ^b^	0.007
(U/L)							
AST	19.42 ± 1.56	25.88 ± 1.30	20.97 ± 1.12	21.55 ± 2.02	22.85 ± 1.68	22.35 ± 2.39	0.064
(U/L)							
HDL-C	0.51 ± 0.09	0.44 ± 0.14	0.54 ± 0.09	0.67 ± 0.20	0.52 ± 0.10	0.53 ± 0.12	0.923
(mmol/L)							
LDL-C	2.57 ± 0.25 ^b^	4.71 ± 0.39 ^a^	2.77 ± 0.48 ^b^	2.53 ± 0.21 ^b^	2.12 ± 0.49 ^b^	2.05 ± 0.24 ^b^	0.005
(mmol/L)							
TG	14.13 ± 1.50 ^b^	21.27 ± 3.05 ^a^	14.16 ± 2.16 ^b^	9.61 ± 1.56 ^b^	12.93 ± 2.60 ^b^	13.27 ± 1.21 ^b^	0.02
(mmol/L)							
TC	3.40 ± 0.21 ^b^	4.78 ± 0.35 ^a^	3.72 ± 0.81 ^b^	3.30 ± 0.23 ^b^	2.93 ± 0.37 ^b^	3.05 ± 0.13 ^b^	0.001
(mmol/L)							

^1^ Values without letters or with the same superscript in the same row are not significantly different (*p* > 0.05), and the superscript values of different lowercase letters were significantly different (*p* < 0.05). Data are presented as the means ± SEM; ^2^ ALT = alanine aminotransferase; AST = aspartate transaminase; HDL-C = high-density lipoprotein cholesterol; LDL-C = low-density lipoprotein cholesterol; TG = triglyceride; TC = total cholesterol; CON: normal control, HFD: addition of 10% of soybean oil to the base diet; addition of 0.01% CDCA, 0.02% CDCA, 0.01% HDCA, and 0.02% HDCA to the HFD, respectively.

**Table 5:** Effects of dietary CDCA and HDCA supplementation on antioxidant capacity of laying hens ^1^.

Items ^2^	CON	HFD	HFD				*p* Value
			0.01% CDCA	0.02% CDCA	0.01% HDCA	0.02% HDCA	
T-AOC	0.48 ± 0.05 ^a^	0.31 ± 0.02 ^b^	0.49 ± 0.05 ^a^	0.44 ± 0.08 ^ab^	0.59 ± 0.04 ^a^	0.52 ± 0.09 ^a^	0.048
(U/mL)							
CAT	2.42 ± 0.45 ^a^	1.15 ± 0.29 ^b^	2.25 ± 0.17 ^a^	2.54 ± 0.05 ^a^	2.24 ± 0.46 ^a^	2.27 ± 0.21 ^a^	0.04
(U/mL)							
MDA	2.34 ± 0.64 ^b^	5.71 ± 0.87 ^a^	2.59 ± 0.49 ^b^	2.58 ± 0.23 ^b^	2.96 ± 0.59 ^b^	3.61 ± 0.27 ^b^	0.009
(nmol/mL)							
T-SOD	55.95 ± 2.50 ^a^	42.84 ± 3.27 ^b^	53.81 ± 1.88 ^a^	57.83 ± 3.60 ^a^	60.34 ± 3.22 ^a^	54.07 ± 1.01 ^a^	0.019
(U/mL)							
GSH	25.28 ± 4.64	12.38 ± 1.93	20.00 ± 2.98	22.22 ± 2.72	20.00 ± 3.09	24.44 ± 2.03	0.097
(μmol/L)							
GSH-PX	483.46 ± 24.44 ^ab^	335.31 ± 19.44 ^c^	427.26 ± 28.41 ^bc^	560.00 ± 43.41 ^a^	372.35 ± 72.66 ^c^	419.56 ± 19.96 ^bc^	0.001
(U/mL)							

^1^ Values without letters or with the same superscript in the same row are not significantly different (*p* > 0.05), and the superscript values of different lowercase letters were significantly different (*p* < 0.05). Data are presented as the means ± SEM; ^2^ T-AOC = total antioxidant capacity; CAT = catalase; MDA = malondialdehyde; T-SOD = total superoxide dismutase; GSH = glutathione; GSH-PX = glutathione catalase; CON: normal control, HFD: addition of 10% of soybean oil to the base diet; addition of 0.01% CDCA, 0.02% CDCA, 0.01% HDCA, and 0.02% HDCA of 10% to the HFD, respectively.

**Table 6:** Effects of dietary CDCA and HDCA supplementation on cytokines of laying hens ^1^.

Items ^2^	CON	HFD	HFD				*p* Value
			0.01% CDCA	0.02% CDCA	0.01% HDCA	0.02% HDCA	
IL-6	35.48 ± 0.73 ^bc^	39.10 ± 0.93 ^a^	38.17 ± 0.94 ^ab^	36.32 ± 0.82 ^abc^	37.02 ± 1.39 ^abc^	34.55 ± 0.452 ^c^	0.019
(pg/mL)							
IL-1β	2.90 ± 0.24 ^b^	3.82 ± 0.23 ^a^	3.39 ± 0.33 ^ab^	2.50 ± 0.26 ^b^	3.18 ± 0.22 ^ab^	2.76 ± 0.33 ^b^	0.044
(pg/mL)							
TNF-α	0.35 ± 0.03 ^b^	0.48 ± 0.04 ^a^	0.30 ± 0.04 ^b^	0.33 ± 0.01 ^b^	0.34 ± 0.03 ^b^	0.25 ± 0.04 ^b^	0.008
(pg/mL)							

^1^ Values without letters or with the same superscript in the same row are not significantly different (*p* > 0.05), and the superscript values of different lowercase letters were significantly different (*p* < 0.05). Data are presented as the means ± SEM; ^2^ IL-6 = interleukin-6, IL-1β = interleukin-1β, TNF-α = tumor necrosis factor-α; CON: normal control, HFD: addition of 10% of soybean oil to the base diet; addition of 0.01% CDCA, 0.02% CDCA, 0.01% HDCA, and 0.02% HDCA to the HFD, respectively.

## Data Availability

The data presented in this study are available upon request from the corresponding author.
